# Probing the link between oestrogen receptors and oesophageal cancer

**DOI:** 10.1186/1477-7819-8-9

**Published:** 2010-02-10

**Authors:** Farhan Rashid, Raheela N Khan, Syed Y Iftikhar

**Affiliations:** 1 Department of Upper GI Surgery, Royal Derby Hospital, Uttoxeter Road, Derby, DE22 3NE, UK; 2 Academic Division of Obstetrics and Gynaecology, University of Nottingham, Uttoxeter Road, Derby, DE22 3DT, UK; 3 Academic Division of Upper GI Surgery, School of Graduate Entry Medicine and Health, University of Nottingham, Derby, DE22 3DT, UK

## Abstract

**Background:**

Human oesophageal carcinoma is considered to be one of the most aggressive malignancies and has a very poor prognosis. The incidence of oesophageal cancer shows a gender bias and is higher in males compared with females, the ratio between males and females varying from 3:1 to 7:1. This sex ratio is not entirely attributable to differences in the prevalence of known risk factors between the sexes. The potential role of oestrogen receptors (ER) in oesophageal cancer has been debated for several years but the significance of the receptors in this cancer remains unknown. Most of the work has been based on immunohistochemistry and has not been validated with other available techniques. The inconsistencies in the published literature on the link between ER expression and oesophageal cancer warrant a thorough evaluation of the potential role of ERs in this malignancy. Even the expression of the two ER isoforms, ERα and ERβ, and its implications for outcome of treatments in histological subtypes of oesophageal tumours is ill defined. The aim of this article is to provide updated information from the available literature on the current status of ER expression in oesophageal cancer and to discuss its potential therapeutic role.

**Methods and Results:**

We performed a comprehensive literature search and analysed the results regarding ER expression in oesophageal tumours with special emphasis on expression of different oestrogen receptors and the role of sex hormones in oesophageal cancer. This article also focuses on the significance of the two main ER subtypes and mechanisms underlying the presumed male predominance of this disease.

**Conclusion:**

We postulate that differential oestrogen receptor status may be considered a biomarker of poor clinical outcome based on tissue dedifferentiation or advanced stage of the disease. Further, if we can establish the importance of oestrogen and its receptors in the context of oesophageal cancer, then this may lead to a new future direction in the management of this malignancy.

## Introduction

**Figure 1:**
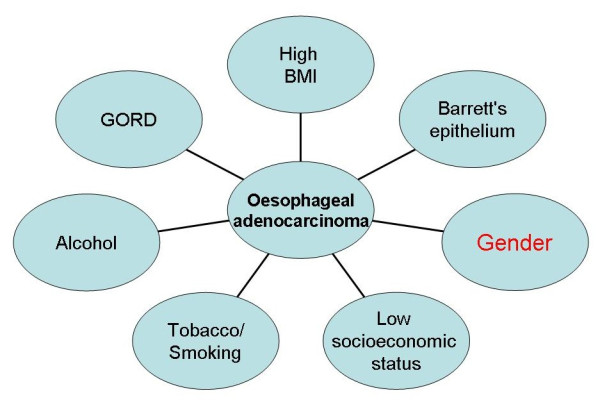
**Aetiological factors for oesophageal adenocarcinoma**.

**Figure 2:**
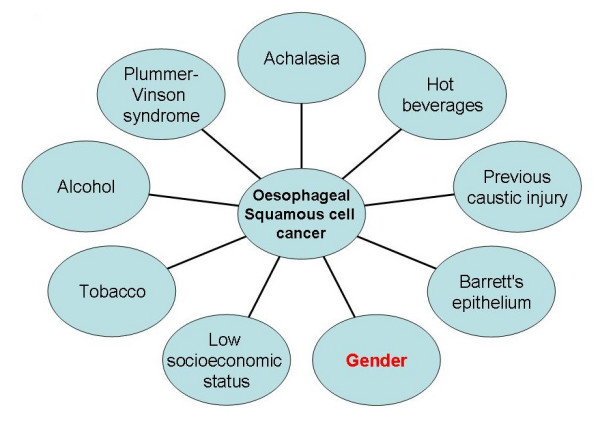
**Aetiological factors for oesophageal squamous cell cancer**.

Human oesophageal carcinoma is the eighth most common type of malignancy in the world [1], with approximately half a million people diagnosed annually worldwide [2]. Over the last three decades, the incidence of oesophageal cancer in many parts of the world has risen significantly [3-7]. The prevalence of the two main histological subtypes of oesophageal cancer, adenocarcinoma (AC) and squamous cell carcinoma (SCC) varies depending upon geographical location [8]. The AC is common in Europe and Australia [9] followed by the USA [8,9], while SCC predominates in Asian countries especially in the far East[10]. The incidence of oesophageal AC in the western world has risen rapidly over several years [11-13] whilst that of SCC has decreased[8], although increasing trends have been observed in Denmark and the Netherlands among men [14]. Carcinogens including dioxins, nitrosamines and polycyclic aliphatic hydrocarbons present in tobacco, processed meats and fried foods along with alcohol consumption and gastrooesophageal reflux disease and others have all been identified as risk factors for oesophageal cancer[15] although contribution of aetiological factors varies amongst histological subtypes of the disease. Figures 1 and 2 depict the risk factors for AC and SCC respectively.

Among the different treatment options, surgical resection is used most frequently to obtain locoregional control and long-term survival [16]. However, because of early tumour recurrence and metastasis, the overall five year survival after resection is around 35% [17,18]

The AC of the oesophagus is predominantly a male disease with a male to female ratio of 6-8:1 [14]. It is also reported that both the AC [19] and SCC [20] of the oesophagus are more common in males than females with a male to female ratio exceeding 3-6:1 or higher in some studies [20-22]. Barrett's oesophagus, identified as a potential risk factor for AC, also occurs predominantly in males with a male: female ratio ranging from 2:1 to 4:1 [23,24]. Lofdhal *et al *have suggested that the sex difference in oesophageal AC does not seem to be explained by differences in risk factor profile of known aetiological agents such as reflux, obesity and tobacco consumption [25].

Badwe *et al *(1994) studied the impact of age and sex on survival after curative resection for carcinoma of the oesophagus with life stable analysis showing a significantly better 5 year survival for women under 49 years of age (35%, CI 24-48) compared with men of the same age (16%.CI 8-27) (P < 0.008)[26]. The gender of the patient was found to be the second most significant determinant of survival (p = 0.002) after lymph node metastasis. These results of better survival benefit for women provides support for the hypothesis that the endocrine milieu in premenopausal women may prevent the micrometastases of the oesophageal malignancy and the consequent improved prognosis for oesophageal cancer [26].

A population-based study by Derakhshan *et al*, has suggested that the increased incidence of oesophageal cancer in females is age-related and occurs postmenopausally [27]. Measuring and correlating systemic sex steroid hormone levels and their interaction with their receptors in pre- and postmenopausal women may help elucidate the age-related incidence in postmenopausal females.

**Figure 3:**
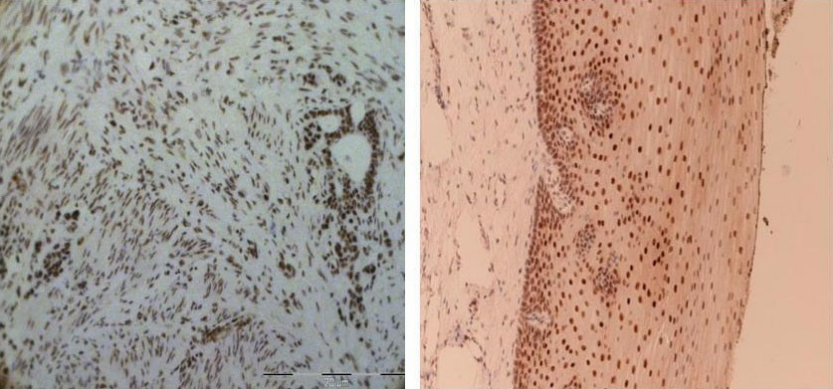
**Immunohistochemical overexpression of oestrogen beta receptors**.

The observation that females appear to have a survival benefit compared to males [28,29] has led us to consider mechanisms through which oestrogens acting via the oestrogen receptor (ER) are implicated in the gender bias thus raising the possibility of using ER status as a positive or negative biomarker of disease outcome. Our recent work on oesophageal cancer has indicated overexpression of immunoreactive oestrogen receptor beta (ERβ) as compared to oestrogen receptor alpha (ERα) and androgen receptors in oesophageal cancer[30] (Figure 3).

## Oestrogen receptors (ER)

**Figure 4:**
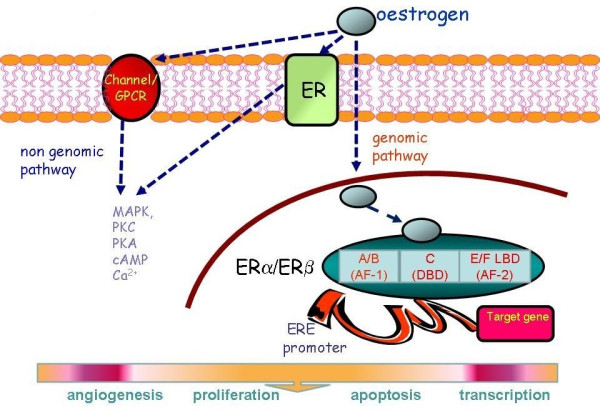
**Pathways of oestrogen action**. The ERα/ERβ is a simplified schematic of the ER. MAPK mitogen activated protein kinase, PKC protein kinase C, PKA protein kinase A, cAMP 3'-5' cyclic adenosine monophosphate, Ca^2+ ^intracellular calcium, DBD DNA binding domain, LBD ligand binding domain.

In addition to their well-documented roles in the reproductive tract, diminished ovarian oestrogen levels are implicated in the development of osteoporosis and the raised risk of cardiovascular disease in postmenopausal women [31]. The actions of oestrogens, a group of C-18 steroids, are mediated principally via ERα and ERβ first cloned from rat prostate [32]. This discovery led to a reappraisal and new perspective on the significance of the ER in health and disease. The ERα and ERβ, isoforms are respectively encoded by two distinct genes (ESR1 and ESR2) located on chromosome 6q25.1 and chromosome 14q22-24[33,34]. The two receptors share common functional domains with a conserved (95% sequence homology) central DNA-binding domain thought to be involved in receptor dimerisation [35] (Figure 4). The ER also possesses two activation function domains AF1 and AF2 [36] with the former interacting with non-ER transcription factors while AF2 contains the ligand binding domain (LBD) [37]. Interestingly, AF1 in ER-β lacks functional activity [36]. Of the natural oestrogens that include oestrone and oestriol, oestradiol-17β (E_2_) has the highest affinity for both ER subtypes.

The ER belongs to the NR3 steroid receptor class of the nuclear receptor superfamily and consistent with the mechanisms of action of this family, it operates via transcriptional regulation to cause downstream changes in gene expression. This follows dissociation of the intracellular ER from chaperone proteins, principally heat shock proteins (eg HSP90) on binding of ligand, thus releasing the ER-complex for attachment to oestrogen response elements located in the promoter region of target genes [31]. The recruitment of coregulators that either activate or repress gene transcription ultimately determines the cellular response. ER functions may also be mediated by non-genomic mechanisms that are predominantly transduced at the membrane and are acute in nature with the physiological response occurring within minutes as opposed to hours [38].

## Expression of ER and other sex hormone receptors in oesophageal cancer

Conflicting data exist on the expression of sex steroid receptors in oesophageal cancer and hence their role in the progression of the disease. Lagergren *et al*., 1998 [21] suggested that in the absence of other known environmental risk factors with a sex distribution which is sufficiently skewed to explain the imbalance in the risk of adenocarcinoma, male predominance might be due to hormonal factors. Either high oestrogen and/or progesterone levels, low testosterone or a combination of these may be the reason why women are apparently protected from developing oesophageal cancer. If the aforementioned presumption is correct, then any treatment that increases oestrogen levels and/or decreases testosterone levels may potentially reduce the risk of developing adenocarcinoma of the oesophagus [21]. However this is a rather simplistic notion when one considers the multifactorial influences and underlying cellular mechanisms that shape development of this disease.

There is limited evidence on progesterone receptor expression in oesophageal cancer although Kalayarasan *et al*., have shown it is absent in both normal epithelial mucosa and oesophageal tumours [39]. With respect to the androgen receptor (AR), Tiffin *et al*., [40] demonstrated focal staining of this receptor in only two of ten patients [40] whilst Awan *et al*., [41] identified AR expression in the stromal component of oesophageal adenocarcinoma [41]. Nuclear and cytosolic AR expression in two newly established human oesophageal carcinoma cell lines (ES-25C and ES-8C) has also been shown [42]. Androgen receptors may be important mediators of oesophageal cancer as shown in studies where oesophageal SCC cell lines underwent enhanced growth when treated with testosterone *in vitro *studies [43,44]. Shinji Tanaka *et al*., are of the opinion that androgens too may play a role in the regulation of gene expression associated with malignant transformation [45].

In studies of the ER expression in oesophageal cancer, Nozoe *et al*., [46] suggested an inverse relationship between ERα and ERβ, in oesophageal squamous cell cancer [46]. Although Kalayarasan *et al*.,, investigating the expression of ER between oesophageal cancer and normal oesophageal mucosa reported no detectable immunohistochemical expression for ERα, the authors propose a positive correlation of ERβ status with tumour dedifferentiation, type and stage [39]. Interestingly, AC showed a higher mean score for ERβ expression as compared with SCC. Furthermore, ERβ positive immunoreactivity in tumour cells increased with dedifferentiation and increasing tumour stage in both types of oesophageal cancer and has prompted suggestions that ERβ status is a potentially useful marker of worsening disease progression [39]. In contrast, to the findings of Kalayarasan *et al*., [39] Tiffin *et al*., [40], identified mild to moderate staining of ER in most of their oesophageal tissue samples but the authors did not discriminate between the ER subtype detected [40]. Given the conflicting evidence on the ERα and ERβ expression in oesophageal cancer, Table 1 summarises the studies performed to date. Cui *et al*., [47] have also examined ER expression but patients in this study had oesophagogastric carcinomas likely originating from the stomach.

**Table 1:** Studies assessing the risk of oesophageal cancer in relation to oestrogen receptors.

	No of patients	M:F	PR	ER-α	ER-β	Histological subtypes of OC	Source & affinity of ER AB	Significance/Dedifferentiation
Kalayarasan *et al*., (India, 2008)[39]	45	3:2 (SCC) 4:1(AC)	0	0	45	AC (n = 15) SCC (n = 30) AC>SCC	Novacastra- ERα- Clone 6F11- ERβ- Clone EMR02-	**ERβ **staining increases with dedifferentiation

Boone J *et al*, (Netherlands,2009)[54]	108 (Tissue Microarray)		0	0		100% s SCC	Dako- M7047	No staining found with **ERα**

Nozoe T *et al*.,(Japan, 2007)[46]	73	10:1	ND	47	21	100% SCC	Santa Cruz- HC-20(α) H-150(β)	**ERα **expression -unfavourable independent prognostic indicator

Tiffin et al., (UK,2003)[40]	20	1:1	ND	ND	ND	ER= +ve (n = 8 AC)	Dako- NS	**Oestrogen receptors **are more important than androgen receptors-require further investigations

Wang L et al., (China,1991)[56]	48		19	Unknown	Unknown	Unknown	Unknown	Gender & grade of tumour were influencing ER expression

Akgun et al., (USA,2002)[49]	31	ND	ND	NA	23	AC	NS- MYEB(β)	AC, BM show higher expression of **ERβ**

Liu et al., (USA, 2004)[55]	33	ND	ND	NA	ERβ 1 = 23/27 ERβ 2 = 22/27 ERβ 3 = 27/27 ERβ5 = 27/27	ACC>Barrett's metaplasia negative for dysplasia	In house- Human ER-β (amino acids 1-12).	**ERβ **subtypes are overexpressed in oesophgael cancer as compared to its precursor lesions

ND: Not determined; PR progesterone receptor; AB: antibody; AC: adenocarcinoma; BM: Barrett's metaplasia; NS: not specified; OC: oesophageal cancer; NA: not applicable

*Tiffin et al[40] (n = 20), Six patients with metaplasia also had positive ER staining

It has been postulated that *in vivo *growth of human oesophageal carcinoma cells mediated via sex hormone receptors is influenced by circulating hormone levels and can be manipulated by systemic oestradiol administration [48]. The effects of various doses of E_2 _on *in vitro *growth of these cell lines has established that one of the cell lines (ES-25C) showed significant inhibition at concentrations of 10^-10 ^and 10^-12 ^mol/l compared with the control, indicating a role for the oestrogen-ER system in growth inhibition of ER+ oesophageal cancer cell by oestradiol-17β [42,48]

In studies following the development of Barrett's to oesophageal cancer, it was found that of 31 patients who underwent oesophagectomy, more than 50% (23/31) were found to have positive staining for ER-β [49]. Among patients with high grade dysplasia,10 out of 11 (91%) showed positivity and this ratio was found to be 8 out of 11 (83%) in low grade dysplasia and 10 out of 15 in patients with Barrett's metaplasia having no dysplasia. The authors of the study conclude that premalignant Barrett's and oesophageal adenocarcinoma display positive ERβ immunoreactivity in a significantly high proportion [49]. Given the inconclusive data on studies of the ER subtype expression in cancer, the poor specificity of earlier antibodies against ERβ has highlighted the need to validate methods and reagents properly. This is especially applicable to studies designed to produce accurate and meaningful outcomes on which to base future applications of ERβ as a prognostic measure.

## ERs and link with other cancers

Mounting evidence supports a role for the ER in halting or promoting cancer progression. Most of our current understanding on the biological significance of ERs in tumourigenesis has emerged from studies of breast cancer and the relationship between the expression of ERα and response to the nonsteroidal antioestrogen, tamoxifen. Oestrogens and ERs also play a vital role in the development or suppression of several other malignancies classified into four main subgroups [50,51] most of which express both ERα and ERβ [50,51]. Specifically, in the Women's Health Initiative study in which a cohort of 16,608 women were randomized into either a hormone replacement therapy (HRT) group or a non-HRT group [52], the risk of colorectal cancer was almost halved in women receiving HRT. Similarly, the association between HRT and risks of oesophageal and gastric cancer was studied in a nested-case control study where a total of 1619563 patients were identified from the General Practitioners Research Database in the UK. The conclusions of this latter study were that HRT leads to a 50% reduction in the risk of gastric adenocarcinoma but there was no relationship between HRT and oesophageal adenocarcinoma [53]. However, only a relatively small number of women with oesophageal cancer (n = 299) [53] were included which may have limited the power of the study. In addition, the lack of such association does not exclude more complex cellular and molecular interactions that are not detectable in this sort of clinical study.

## Mechanisms underlying altered ER expression and activity in oesophageal cancer and therapeutic implications

Based on the available yet rather scarce literature on the potential association of ER receptor expression with oesophageal cancer [39,40,46,49,54-56], some of the mechanisms underlying ER and E_2 _interactions in oesophageal cancer are based on studies of other cancers in which ERs have been implicated and are briefly discussed below:

## Differential ERα and ERβ expression and the ERα:ERβ ratio in cancer

In many cancers, ERα appears to be instrumental in promoting cell proliferation. However, recent studies have suggested that both mRNA and protein levels of ERβ may have greater significance in certain cancers. A loss of ERβ expression is observed in colorectal [57,58], prostate [59] and breast [60] carcinoma that all express high levels of ERβ in normal tissues [60,61]. Decreased ERβ levels are associated with improved disease outcomes and longer term disease-free survival in malignant mesothelioma attributed to the antiproliferative effects of ERβ[62]. The beneficial effects of HRT in colon cancer, which expresses very little ERα in normal colon, are likely to be mediated by downregulation of ERβ [58]. Evidence supports a possible protective role for ERβ in prostate cancer where a loss of ERβ expression accompanied the development of prostate cancer [59]. Interestingly, the small number of cancers that continued to express ERβ were positively correlated with a higher rate of relapse [59]. In malignant mesothelioma, attenuated ERβ expression appears to be an independent indicator of improved prognosis and survival [62]. Yet in tissues expressing both isoforms of the ER at comparable levels, the growth inhibitory effects of ERβ are less obvious thought to be due to the lack of AF1 ERβ activity. ERβ is known to bind to and suppress ERα function [63,64] thereby demonstrating inverse biological activity.

Activation of ER-α and ER-β involves the formation of dimers and as the two isoforms are coexpressed in many cell types, receptors may exist as ERα (αα) or ERβ (ββ) homodimers or as an ERαβ (αβ) heterodimer [65]. Homo- and heterodimerisation may also introduce diversity of tissue and cell-specific functions.

In oesophageal cancer, it is likely to be the relative abundance of ERα:ERβ, dominance of one ER dimer over another and their roles in many of oestrogen's nonendocrine functions that likely contribute to disease onset and severity.

## Phosphorylation and ligand-independent activation of ER

Gene transcription via ER may proceed indirectly without binding of native ligand to oestrogen response elements involving instead protein-protein interactions. The most prominent for ERα appear to be the transcription factors, specificity protein (SP-1) and nuclear factor kappa b (NFκB), the proinflammatory transcription factor. The activator protein-1 (AP-1) complex of Jun/Fos hetero- or homodimers is a key regulator of cell proliferation with one of the target genes identified as cyclin D1. Depending on the whether ERα or ERβ is activated, the AP-1 complex acts in a reciprocal fashion to stimulate or inhibit cell proliferation. Ligand-independent activation may also determine the phosphorylation status of ERα which occurs principally at serine residues in the AF1 domain (Figure 4) [31]. Phosphorylation of ER may also occur via a plethora of bioactive mediators that include growth factors, cytokines and enzymes and has been linked with hormone-independent growth, loss of cell-cell adhesion and angiogenesis.

## Proliferation and apoptosis

The mitogenic and growth-promoting actions of oestrogens in target tissues are well-established and are achieved in part by increased transcription of cell-cycle genes via ERα. However, in certain cancers where ERβ is considered protective, antiproliferative effects are achieved by cell cycle growth arrest for example by down-regulation of the cyclin D1 (CCND1) gene thereby preventing cellular progression from the G1 to S-phase of the cell cycle. ERβ may also inhibit gene transcription induced by ERα. These lines of evidence have led to the suggestion that ERβ acts as a tumour suppressor gene and is supported by findings that show localisation of ERβ to chromosome 14q is shared by other tumour suppressor genes that exert protective effects in prostate and ovarian cancer [61]. Regression of tumours and improved survival may be achieved by inhibition of cell proliferation as discussed or alternatively by increased apoptosis as has been observed in prostate cancer. Apoptosis involves a series of cellular events involving loss of membrane gradients, DNA fragmentation and caspase activity. In cancers, such as malignant mesothelioma [62], ERβ appears to be proapoptotic thus enabling it to destroy malignant cells whilst ERα has antiapoptotic activity which underpins its role in normal and abnormal cell proliferation.

## Epigenetic modifications

Tumourigenesis may be triggered by epigenetic changes that involve modifications to chromatin structure including DNA methylation and altered histone acetylation thus causing downstream changes in gene expression [66]. Studies in prostate and breast cancer [67] have demonstrated hypermethylation of the ERβ promoter with subsequent silencing of ERβ expression but no evidence yet exists for altered ER methylation in oesophageal cancer.

## Circulating oestrogen levels

In premenopausal women, ovarian E_2 _levels are high, largely attributable to the aromatisation of testosterone to oestradiol-17β within the ovary. For postmenopausal women in whom ovarian E_2 _levels are reduced, oestrone is the most abundant oestrogen formed from its precursor, androstenedione. Peripheral aromatization of oestrone from androgens in adipose tissue is one mechanism whereby circulating oestrogen levels may be increased, perhaps explaining in part the gender selectivity of oesophageal cancer. Although it is unlikely, that oestrogen levels will rise to those present in premenopausal women, given the lower affinity of oestrone for ERα, oestrone may still have the capability to confer oestrogenic effects but with less potency. To date, it is not known whether aromatase is produced by the oesophagus but if it is, then it may be factors such as the ratio of local oestrogen to androgen production as well as the form of oestrogen produced (oestrone, oestradiol, oestriol) that may underlie gender bias and the increased risk of postmenopausal women and males to oesophageal cancer compared with their premenopausal females. More scientifically robust studies as proposed by Hogan *et al*., are needed [68].

## Therapeutic relevance of ERs to oesophageal cancer

Therapeutic modalities currently in place for modulation of ER activity include selective oestrogen receptor modulators (SERMs) e.g. tamoxifen which exhibits oestrogenic activity depending upon the target tissue. Tamoxifen, acts as an agonist in bone and the cardiovascular system in postmenopausal women but as an antagonist in the breast of premenopausal women where it has revolutionized breast cancer treatment in the form of adjuvant endocrine therapy [69]. In the same way, another popular SERM, raloxifene is used in osteoporosis to improve bone mineral density where it exhibits greater agonist activity [69]. The variation in the response of a particular SERM either as an agonist or antagonist depends on several factors. Thus, once a SERM attaches to the ER, a specific conformational change in the receptor is induced which determines which corepressors and/or coactivators are recruited to the promoter. Based upon these factors, tamoxifen recruits a coactivator complex to oestrogen regulated genes in endometrial cells whilst it recruits a corepressor complex to the same gene in breast cancer cells [70]. There is a paucity of information in relation to the role of phytooestrogens, albeit of lower potency at the ER but, appear to exhibit greater selectivity for ERβ over ERα [71] An effect of environmental oestrogens and ERs in the pathogenesis of oesophageal cancer has not been reported to date but may introduce another level of complexity in the contribution of ER in the aetiology of this disease.

## Potential for future research

Most patients with oesophageal cancer present late with inoperable disease. Despite recent advancement in surgical and oncological treatment, the five year survival after oesophagectomy is about 25% [72,73] hence new means of predicting disease onset and treating oesophageal cancer in order to improve outcomes need to be explored. From the limited number of investigations reported for ER expression in oesophageal cancer, the varied experimental design of these studies, different antibodies used and few other techniques to confirm these findings, it is too early to draw definitive conclusions regarding the future therapeutic utility of ERs in oesophageal cancer. However, evidence presented herein indicates that the presence of ERs appears to have greater significance than other sex steroid receptors while three studies report relatively raised ERβ expression with oesophageal tumour dedifferentiation. Although we are some considerable way off from understanding the apparent paradox of increased ER expression in oesophageal cancer and a seemingly better prognosis in women, a concerted research effort is required in order to determine relative levels of ERα:ERβ according to gender and age, ER expression patterns with disease progression, modulation of oestrogen production and the role of environmental and phytooestrogens, by immunochemical, molecular and functional assays. The use of powerful experimental techniques such as gene microarrays, chromatin immunoprecipitation to investigate transcriptional regulation of ER and silencing of ER subtypes using siRNA methods to tease apart the complexity of the disease process will provide us with deeper insight into underlying mechanisms at play.

Although the two histological subtypes AC and SCC, vary in their origin, aetiology and incidence, the strong male predominance of oesophageal cancer highlights the importance of further investigation regarding oestrogen receptors and ER pathways, as also agreed by a recently published review by Chandanos et al [74]. If conclusive evidence of a role for oestrogen and its receptors s obtained, then this paves the way for the development of a new diagnostic biomarker in early diagnosis and treatment of this disease. Published studies provide only a hint of the possible use of sex hormone therapy for managing oesophageal cancer. Nonetheless, if a role for ERs in oesophageal cancer is proven this could potentially lead to new and revolutionary approaches in the form of hormonal therapy to treat oesophageal cancer.

## Search strategy and selection criteria

Information for this personal review was obtained by searches between March 1978 to October 2009 of Pubmed using the following key words: 'oestrogen receptors', 'sex hormones', 'oesophageal cancer' and 'oestrogen'. Papers or abstracts published in English were included. All authors reviewed original articles and reviews for relevance and included all pertinent studies in the preparation of the manuscript. We have also considered the bibliographies of the selected articles for the pertinent citations.

## Competing interests

The authors declare that they have no competing interests.

## Authors' contributions

FR and RNK have reviewed the literature. FR has performed the laboratory based work. RNK and SYI provided the supervision. FR wrote the manuscript. RNK and SYI edited the manuscript. All authors contributed to the manuscript, and all read and approved the final version
